# A pilot program in rural telepsychiatry for deaf and hard of hearing populations

**DOI:** 10.1016/j.heliyon.2016.e00077

**Published:** 2016-03-11

**Authors:** Teresa Crowe, Suni Jani, Sushma Jani, Niranjan Jani, Raja Jani

**Affiliations:** a Hall Memorial Building, Gallaudet University, 800 Florida Avenue, NE Washington, DC 20002-3695, United States; b Child and Adolescent Psychiatry, Massachusetts General Hospital/McLean Hopsital, Harvard Medical School, Boston, MA 02144, United States; c Community Behavioral Health, 426 Dorchester Ave, Cambridge, MD 21613, United States; d Community Behavioral Health, The George Washington University School of Medicine, 821 Eastern Shore Drive, Salisbury, MD 21804, United States; e Touro College of Medicine, 230 W 125th Street, New York, NY 10027, United States

**Keywords:** Mental health, Health services, Health systems

## Abstract

**Background:**

Access to mental health care in deaf communities is limited by cultural considerations, availability of translators, and technological considerations. Telepsychiatry can mitigate the deaf community’s lack of access to care by allowing for deaf individuals in remote communities access to care with facilities that cater to their needs.

**Methods:**

Community Behavioral Health, Arundel Lodge, and Gallaudet University worked in conjunction to test three hypotheses:

1. Telepsychiatry will be as effective as traditional face-to-face psychotherapy with deaf adults who have chronic mental illness.
2. Patients living in remote locations will report an improvement in accessibility to mental health services.
3. Patients who receive telepsychiatry will report a comparable level of satisfaction of services to those receiving traditional services.

The patient sample consisted of 24 participants, 13 women, 11 men. Telepsychiatry sessions were scheduled based on each patient’s individual treatment plan against a control group who saw their providers face to face.

**Results:**

The telepsychiatry and in-person groups were slightly different at baseline. Analysis of the data revealed no significant difference in coping abilities and psychiatric symptoms between those receiving face-to-face psychotherapy and those receiving telepsychiatry.

**Interpretation:**

The quality and outcome of care was equal to in-person for the telepsychiatry in deaf patients. Since telepsychiatry does not compromise the quality of care, it is a good means of reaching out to members of the deaf community that cannot readily access mental health resources that meet their needs.

## 1 Introduction

Deaf Communities are distinctive cultural groups with diverse cognitive, social, and emotional developments and presentations [1, 2]. They are the groundwork for social intercourse for individuals with a unique culture comprising of sign language as a primary form of communication. These are isolated communities with limited access for hearing individuals, which is of particular concern in relation to mental illnesses and their treatment. Several studies have demonstrated high rates of mental health problems in deaf adults [3, 4, 5, 6, 7] with additional studies showing twice the prevalence of emotional and behavioral problems in deaf children [8, 9, 10, 11, 12, 13].

Assessment and treatment of telepsychiatry for the deaf is complex. When mental health services can be provided for deaf and hard of hearing individuals, there are numerous sociocultural considerations that present in the therapeutic relationship between clinician and patient. These considerations include the communication environment in a family, [14] stress or bullying in a school environment, [15] perceived emotional availability and coping styles of parents, [16, 17, 18] additional disabilities,[19, 20, 21], and source of income [22, 23]. These realities all contribute to a unique picture of mental illness in an often overlooked culture.

Most common mental illnesses for the deaf have the same prevalence rate as the regular population, except for higher rates of somatization and anxiety disorders [3, 5, 6]. There is a higher prevalence of impulse control disorders, autism, learning disabilities, and pervasive developmental disorders in deaf populations but these are often overlooked as many symptoms are attributed to deafness [24, 25, 26, 27]. Deaf individuals have increased substance use [28], insomnia [35], and violent crimes [31, 32, 33, 34] compared to the hearing populations. Psychotic illnesses are underreported and unidentified in the deaf population as thought disorganization is difficult to ascertain [29, 30].

Deaf patients may struggle to engage with providers due to limited health literacy, sub-optimal past experiences that affect trust [36], and clinics which do not have a range of disciplines in culturally sensitive sign language interpretation [37, 38, 39, 40, 41]. Reaching a provider with access to an interpreter is an arduous task for most deaf individuals [42]. Even with good outcomes from deaf specialists, there is a shortage in their availability which remains an ongoing barrier to care access for many deaf individuals [43]. The scarcity of outpatient specialists equipped to manage the deaf population has resulted in longer inpatient psychiatric hospitalizations as there was no guarantee for outpatient follow up for deaf individuals and placement proved to be a difficult task for hospitals [44, 45].

Mental health services for deaf adults who use American Sign Language are sparse in comparison to those offered to hearing adults [50]. This may be partly due to the fact that providers with knowledge of deaf culture and fluency in American Sign Language are few. Deaf people who live in rural environments are especially affected by this problem. Deaf adults and deaf children who receive mental health services should work with competent providers who are able to communicate effectively [51, 52]. Simply using an interpreter in therapy sessions is not an optimal accommodation, though it may be the only option available when there are no culturally competent clinicians nearby. An optimal therapeutic session is when the clinician herself has an understanding of the cultural context within which the patient lives [52]. In addition, a culturally competent clinician can understand the social impacts a deaf person faces when interacting within a hearing environment. An interpreter, whose role is to simply translate sign language into the spoken word (and vice versa), may not have the clinical skills needed to interpret underlying meaning in a holistic perspective. Therefore, a clinician unable to communicate with her patient directly may not receive the necessary information needed to assess underlying nuances.

Deaf individuals typically already have technological expertise with videoconferencing because they often use videophones as their telephone [51]. Therefore, pilot telepsychiatry programs for deaf populations were briefly tried in North and South Carolina in 1997 [53, 55] with an existing program in Illinois in 2014 [54]. These have been successful ventures. Unfortunately, few agencies offer telepsychiatry for deaf patients. Thus, there are few research findings comparing the effectiveness of telepsychiatry health services [52]. In one study cited by Gournaris (2009), findings suggested that there is no significant different between face-to-face therapy and videoconferencing with deaf adults receiving treatment but further research was required [51].

In 2008, the Maryland Advisory Council of the Deaf and Hard of Hearing Mental Health subcommittee of the Department of Health and Mental Hygiene met to address problems with access to mental health services by deaf consumers [56]. They suggested that telepsychiatry is a viable option for deaf consumers. The service offers a solution to both the lack of services in remote geographic areas and the lack of culturally and linguistically competent professionals. Their initiative and funding assisted with the realization of this project to address the unique mental health disparities of the deaf and hard of hearing populations.

This report attempts to study several hypotheses with regards to telepsychiatry in rural deaf populations. The first is that telepsychiatry will be as effective as traditional face-to-face psychotherapy with deaf adults who have chronic mental illness. The second hypothesis is patients living in remote locations will report an improvement in accessibility to mental health services. The third hypothesis is that patients who receive telepsychiatry will report a comparable level of satisfaction of services to those receiving traditional services.

## 2 Methods

### 2.1 Approval process

The Institutional Review Board at Gallaudet University approved this study. The design for this project is a pre- post- test, group comparison design. Researchers met with potential participants to explain the study. Once consent was obtained, the participants were grouped into one of two groups: a) control group consisting of those receiving traditional face-to-face psychotherapy at the clinic, and b) experimental group consisting of those receiving telepsychiatry. The researchers collected baseline data on the outcome variables which were measured in a time series fashion at 1 year, 2 years, and 3 years or when the patient terminates therapy.

### 2.2 Participants

#### 2.2.1 Recruitment

Because the deaf population on the eastern shore of Maryland is dispersed throughout a large rural area, the researchers used a combination of informal and formal strategies to recruit participants. The researchers contacted a community service agency that provided services to deaf individuals to begin initial recruitment. They then held community meetings and invited deaf individuals and mental health providers to learn about the project. The researchers also met with individual providers, such as vocational rehabilitation counselors and private practitioners who may serve deaf individuals. The researchers distributed specially designed recruitment materials to agencies and organizations whose staff may interact with deaf individuals, such as hospitals, private clinics, the National Association of the Deaf, the Governor’s Office for the Deaf and Hard of Hearing, and other community service agencies.

### 2.3 Locations

Two community health clinics provided locations for this study. The first was Arundel Lodge, Inc., an organization that began providing psychiatric rehabilitation services to adults with serious mental illness since 1975. An integral part of Arundel Lodge is the programs that serve deaf individuals with severe and persistent mental illnesses. Sensitivity to deaf culture, the use of sign language, and integration into the larger deaf community are key aspects of the residential program, day program, and outpatient mental health clinic that provide services to deaf adults.

The second location was Community Behavioral Health (CBH), an outpatient mental health clinic providing psychotherapy, medication management, and psychiatric rehabilitation services to adults, children, and adolescents with a wide variety of psychiatric illnesses on the rural Eastern Shore of Maryland since 2012. The clinic also began certified addiction services to provide higher intensity individualized care to patients with co-morbid conditions of substance use and mental health disorders.

In partnership with Arundel Lodge and Gallaudet University, CBH expanded to serve deaf and hard of hearing populations with mental illnesses. Counseling and medication management were possible through a licensed clinical social worker and psychiatrist with the aid of an interpreter in face-to-face sessions but to improve outreach, the program applied for a grant to receive telepsychiatry equipment.

#### 2.3.1 Demographics

The sample was comprised of 24 deaf or hard of hearing participants, 13 women (54.2% of the sample) and 11 men (45.8%). The ages of participants ranged from 23 years old to 83 years old (M = 46.38, SD = 14.50, Median = 47.50). Twenty-two participants (91.7%) prefer to communicate primarily using American Sign Language. The most frequent psychiatric diagnosis for the participants was a mood disorder (N = 20, 80% of the sample); three were diagnosed with a psychotic disorder (12%); two were diagnosed with an anxiety disorder (8%). Eleven participants (45.8%) received traditional face-to-face psychotherapy; 13 participants (54.2%) received psychotherapy via telepsychiatry using videoconferencing equipment. All participants saw their psychiatrists face-to-face in a mental health clinic. The majority of participants (N = 20, 83.3% of the sample) had previously received services from an outpatient clinic. Less than half of the participants (N = 11, 45.8%) received rehabilitative services, such as day program or psychiatric rehabilitation program (PRP). See Table 1 for additional demographics of the sample.

### 2.4 Intervention

**Fig. 1:**
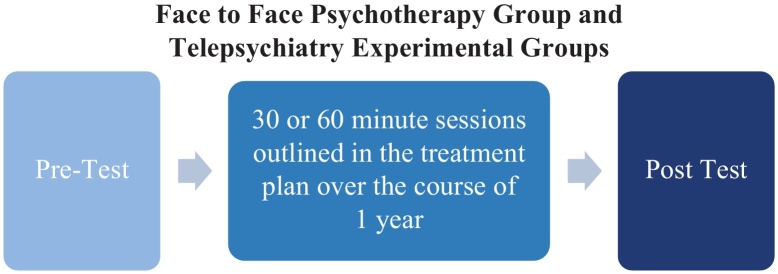
Study flow chart.

Telepsychiatry sessions were scheduled on a regular weekly or biweekly basis depending upon the patient’s individual treatment plan (ITP). Sessions were either 30 min or 60 min as outlined in the patient’s ITP. The therapist is fluent in American Sign Language, the primary language of the patients served. The therapist was based at a community mental health clinic in a private office with videoconferencing and/or videophone technology. Patients went to community-based outpatient mental health clinic on the eastern shore to use the videoconferencing equipment to receive psychotherapy (Fig. 1).

### 2.5 Measures

The assessments and follow ups will include the following outcome measures:

1. Maryland’s Consumer Perception of Care Survey (2010) which includes:
  • Demographic variables: Age, gender, race, education, living situation;
  • Benefits of mental health services received; and
  • Patient satisfaction of services.
2. Maryland Outcomes Measurement System Adult Questionnaire (OMS). This questionnaire is used by the Department of Health and Mental Hygiene as part of its state tracking system for individuals who receive mental health services. The questionnaire is a 49-item measure of progress in multiple life domains, including employment, housing, psychiatric symptoms and functioning, substance use, legal system involvement and general health.
3. Coping Abilities. This was a 13-item subscale created from the OMS instrument. See Table 2 for a more detailed description.
4. Psychiatric symptoms: This was a 11-item subscale created from the OMS instrument. See Table 2 for a more detailed description.

**Table 2:** Coping skills and psychiatric symptoms questionnaire.

Subscale Items for Coping Abilities	Subscale Items for Psychiatric Symptoms
• I do things that are meaningful to me. • I am able to take care of my needs. • I am able to handle things when they go wrong. • I am able to do things that I want to do. • My symptoms bother me. • Difficulty coping with problems in your life. • Difficulty concentrating. • How well do you get along with people in your family? • How well do you get along with people outside your family? • How well do you get along in social situations? • How often do you feel close to another person? • Do you feel like you had someone to turn to if you need help? • How confident do you feel in yourself?	• How often do you feel sad or depressed? • How often do you think about ending your life? • How often do you feel nervous? • How often do you have thoughts racing through your head? • How often do you think you have special powers? • How often do you hear voices or see things? • How often do you think people are watching you? • How often do you think people are against you? • How often do you have mood swings? • How often do you feel short-tempered? • How often do you think about hurting yourself?

## 3 Results

### 3.1 Baseline comparisons of mental health outcomes by type of therapy

Prior to the intervention, the two groups, those receiving traditional psychotherapy and those receiving telepsychiatry, were examined. The mean score on the coping subscale was 26.10 (SD = 7.63) out of a possible score of 65 (higher numbers indicated more difficulty) for those who received face-to-face therapy compared to the scores of those who received telepsychiatry (M = 27.56, SD = 12.10). The mean score on the psychiatric symptoms subscale was 18.80 (SD = 4.34) out of a possible score of 60 (higher numbers indicated more severe symptoms) for those who received face-to-face therapy compared to the scores of those who received telepsychiatry (M = 17.50, SD = 7.80).

T-tests compared mental health outcomes between those who received traditional face-to-face psychotherapy with those who received telepsychiatry on two dimensions, coping abilities and overall psychiatric symptoms. The results of baseline data revealed no significant difference on coping abilities (t = −0.317, 17, p = 0.755) and psychiatric symptoms (t = 0.469, 20, p = 0.7644) between those receiving face-to-face psychotherapy and those receiving telepsychiatry. Thus, the two groups were similar in terms of psychiatric symptoms and coping abilities prior to the intervention.

### 3.2 Group comparisons across variables by type of therapy

In order to compare variables between those receiving face-to-face therapy and those receiving telepsychiatry, cross-tabulations across variables were calculated. Table 3 provides summary data for specific variables of those who receive face-to-face therapy and those who receive telepsychiatry health. The majority of participants resided in residential rehabilitation programs (73.33% F2F; 61.54% TMH) and reported to be in good to excellent health. The majority of participants were diagnosed with mood disorders (80% for F2F; 84.62% for TMH). Only one individual reported being arrested within the past six months. In the TMH group, 69.23% reported being employed compared with 46.67% of the F2F participants. Nearly all of the TMH participants (92.31%) reported that they did not smoke cigarettes compared to 66.67% of the F2F group. All of the F2F participants attended an outpatient mental health program compared to 69.23% of the TMH group. See Table 3 for additional details of comparison between the F2F group and the TMH group.

A t-test comparison of coping abilities among those who received face-to-face psychotherapy vs. telepsychiatry revealed a non-significant difference (t = −1.182, 14, p = 0.072).

A t-test comparison of psychiatric symptomology among those who received face-to-face psychotherapy vs. telepsychiatry revealed a significant difference (t = 4.037, 13, p < 0.0001). The post-intervention mean score for psychiatric symptomology for those receiving traditional psychotherapy was almost double the score (M = 20.30, SD = 4.69) compared to the mean of those receiving telepsychiatry (M = 11.60, SD = 0.89).

Refer to Table 4 for data related to perceived convenience and accessibility of services.

The majority of participants receiving face-to-face psychotherapy, 81.82% (N = 9) reported being satisfied with services compared with 100% (N = 6) satisfaction of those receiving telepsychiatry.

## 4 Discussion

The results of this pilot program revealed promising trends in the arena for telepsychiatry for the deaf and hard of hearing, despite the small population. Like many previous studies [52, 53, 54, 55, 56] that reflect the efficacy of telepsychiatry as a comparable intervention with similar outcomes to face to face therapy, this studies shows that even with the added measure of linguistic and culturally competent mental health trained interpreters added to the intervention, it is possible for deaf and hard of hearing patients in remote regions with access to care barriers to receive appropriate mental health services with the aid of telepsychiatry. The potential for replicating this study is high due to the use of outcome measures for symptoms identical to hearing patients. Furthermore, the study yielded improved cultural competence of psychiatrists with no ASL training through increased exposure to deaf and hard of hearing patients with the aid of an interpreter and access through tele-health technology.

Both groups reported a high level of satisfaction with service provision. They were happy with the convenience and accessibility of services. This is particularly important in a rural area where consumers may not have a number of options to receive services. Often providers who offer services are limited; mental health providers who are fluent in American Sign Language are virtually non-existent in rural areas.

The participants of this study had psychiatric symptoms that were significantly less than those receiving face-to-face services. One explanation may be that there is a smaller sample size of deaf and hard of hearing individuals as compared to the number of hearing individuals; thus, the variety and severity of disease will not be as comparable in these populations in general. Finally, telepsychiatry may not be feasible if a patient’s functioning level makes it difficult to use video-based services. Therefore, those individuals capable of receiving psychiatric services were higher functioning. Compared to the general United States population, this study’s participants on average showed a greater prevalence of mood, psychotic, and anxiety disorders. The Maryland Department of Health and Mental Hygiene (DHMH) does not track specific data for the hard of hearing [58]. Statistics obtained from Gallaudet University show that 13% of the United States is deaf or hard of hearing, with the largest portion being aged 65 and over [58]. In Maryland the total hearing impaired population is 55,235, representing 1.5% of the Maryland population [58]. The National Institute of Mental Health (NIMH) reports that on average lifetime prevalence for any mood disorder in the United States is 14%, whereas the Parent’s Checklist (PCL) developed specifically for deaf children reports that 77% of deaf children as having behavioral disorders [57]. In an alternate survey, the Child Behavior Checklist (CBCL), 40% of hearing impaired children were reported to have clinically significant problems [56].

There are limitations to this study, including the fact that the patients enrolled in this study were also enrolled in psychiatric rehabilitation programs, specialized services that are uncommon in many rural communities for hearing patients with even less for the deaf and hard of hearing worldwide. The majority of participants in both groups were diagnosed with mood disorders. Studies examining the effectiveness of TMH services with individuals who have anxiety or psychosis are not as prevalent as those studying individuals with mood disorders. In fact, studies examining TMH with deaf and hard of hearing individuals in general are scarce. Because the sample size was small, additional post-hoc comparisons between those with mood disorders and those with psychosis or anxiety were not feasible. Investigators of future studies may want to examine the impact of diagnosis on service delivery. An additional limitation of the study design involved difficulty in information exchange between Arundel Lodge and Community Behavioral Health due to non-compatible EMRs, which created extra measures to improve paperwork collaboration in order to ensure the patients’ treatment team shared a common understanding of treatment planning and prognosis. Future studies are encouraged to have identical or communicating EMRs. Though these are some limitations with the data from this study, the researchers have determined a large cohort study of this nature is unlikely as the deaf and hard of hearing with mental illnesses are specialized groups in high population regions with even less prevalence in rural regions. Additionally, the success of this study was highly dependent on the cultural competence of interpreters in mental health terminology and symptomatology in addition to ASL. Replication of these studies in other regions will be dependent on effective interpreters and communicative EMRs though the treating team does not require knowledge in specific presentations of mental illness in the deaf and hard of hearing.

Ultimately, the anticipated impact of this program is a method of improving access to psychiatric care for people who are deaf and hard of hearing in areas with limited mental health resources. This pilot program illustrated telepsychiatry is a way to improve healthcare provision for less prevalent conditions in obscure regions. Endeavors such as this one will also demonstrate outcomes and common issues for the deaf and hard of hearing that can be used to guide treatment planning, create specialized psychiatric rehabilitation programs, and improve awareness through preventive measures. Furthermore, increasing exposure to the deaf and hard of hearing with the assistance of interpreters will create mental health and linguistic cultural competence. A culturally sensitive evaluation to create an accurate diagnosis with appropriate treatment involves a thorough assessment of language modality and language fluency, deafness/audiological history, and cultural identification [46, 47, 48]. Signing deaf patients seem reasonably satisfied with telepsychiatry, although staff needs to be familiar with such technology to encourage broad adoption to address the needs of deaf patients in underserved and rural populations [49, 50]. Working towards improving access of care to the deaf and hard of hearing also improves mental health care providers’ and a community’s confidence in treating a wide variety of populations.

## Declarations

### Author contribution statement

Teresa Crowe: Conceived and designed the experiments; Performed the experiments; Analyzed and interpreted the data; Contributed reagents, materials, analysis tools or data; Wrote the paper.

Suni Jani: Analyzed and interpreted the data; Wrote the paper.

Sushma Jani, Niranjan Jani: Performed the experiments; Analyzed and interpreted the data; Contributed reagents, materials, analysis tools or data.

Raja Jani: Wrote the paper.

### Competing interest statement

The authors declare no conflict of interest.

### Funding statement

This study was supported by the Maryland Department of Mental Health and Hygiene.

### Additional information

No additional information is available for this paper.

## Figures and Tables

**Table 1:** Demographics of the sample.

Variable	N	Percent of Sample
Race		
Caucasian or White	12	50.00%
African-American or Black	10	41.70%
Latino	1	4.20%
Biracial	1	4.20%
Marital Status		
Never married	17	70.80%
Married or living together	4	16.70%
Widowed	3	12.50%
Highest Level of Education		
Finished high school or GED	17	70.80%
Some college (no degree)	4	16.70%
Did not graduate high school	2	8.30%
Some vocational school or training	1	4.20%
Current Residence		
Residential Rehabilitation Program (group home)	16	66.70%
Private Residence	7	29.20%
Assisted Living	1	4.20%

**Table 3:** Group comparisons of specific patient variables for face-to-face and telepsychiatry services.

Variable	F2F (N)	F2F% of sample	TMH (N)	TMH% of sample
Current residence				
Private home	3	20	5	38.46
Residential rehabilitation program, group home	11	73.33	8	61.54
Assisted living	1	6.67	0	0.00
Satisfaction with current living situation				
Satisfied	11	73.33	11	84.62
Neutral	2	13.33	2	15.38
Unsatisfied	2	13.33	0	0.00
Homeless in the past 6 months				
No	15	100.00	12	92.31
Yes	0	0.00	1	7.69
Satisfaction with recovery				
Satisfied	14	93.33	10	76.92
Neutral	1	6.67	2	15.38
Unsatisfied	0	0.00	1	7.69
Arrested within the past 6 months				
Yes	1	6.67	0	0.00
No	14	93.33	13	100.00
Incarcerated within past 6 months				
Yes	0	0.00	0	0.00
No	15	100.00	13	100.00
Currently employed				
Yes	7	46.67	9	69.23
No	8	53.33	4	30.77
Smoke cigarettes				
Yes	5	33.33	1	7.69
No	10	66.67	12	92.31
General health				
Excellent or very good	7	46.67	4	30.77
Good or fair	8	53.33	7	53.85
Poor	0	0.00	1	7.69
Primary diagnosis				
Psychotic disorder (e.g., schizophrenia spectrum disorders)	1	6.67	2	15.38
Mood disorder (e.g., depressive disorder with or without psychosis)	12	80.00	11	84.62
Anxiety disorder (e.g., obsessive-compulsive disorder, generalized anxiety disorder)	2	13.33	0	0.00
Attended an outpatient mental health program				
Yes	15	100.00	9	69.23
No	0	0.00	4	30.77
Received psychiatric rehabilitation services (day program or PRP)				
Yes	14	93.33	1	7.69
No	1	6.67	12	92.31
Received residential services (group home)				
Yes	12	80.00	8	61.54
No	3	20	5	38.46

**Table 4:** Convenience and accessibility of services for face-to-face groups and telepsychiatry health group.

Variable	F2F (N)	F2F % of sample	TMH (% of sample)	TMH (N)
Services provided at convenient time				
Always	11	100	6	100
Sometimes	0	0	0	0
Not at all	0	0	0	0
Received all services patient felt were needed				
Always	10	90.91	6	100
Sometimes	1	9.09	0	0
Not at all	0	0	0	0

*Totals for services received were calculated only for those who had received services previously. Many of the patients in rural areas had never received services of any kind due to the lack of accessibility.
